# Impact of provision of cardiovascular disease risk estimates to healthcare professionals and patients: a systematic review

**DOI:** 10.1136/bmjopen-2015-008717

**Published:** 2015-10-26

**Authors:** Juliet A Usher-Smith, Barbora Silarova, Ewoud Schuit, Karel GM Moons, Simon J Griffin

**Affiliations:** 1 The Primary Care Unit, University of Cambridge School of Clinical Medicine, Cambridge, UK; 2 MRC Epidemiology Unit, University of Cambridge, Institute of Metabolic Science, Cambridge, UK; 3 Julius Centre for Health Sciences and Primary Care, University Medical Centre Utrecht, Utrecht, The Netherlands; 4 Stanford Prevention Research Center, Stanford University, Stanford, USA

**Keywords:** PREVENTIVE MEDICINE, CARDIOLOGY

## Abstract

**Objective:**

To systematically review whether the provision of information on cardiovascular disease (CVD) risk to healthcare professionals and patients impacts their decision-making, behaviour and ultimately patient health.

**Design:**

A systematic review.

**Data sources:**

An electronic literature search of MEDLINE and PubMed from 01/01/2004 to 01/06/2013 with no language restriction and manual screening of reference lists of systematic reviews on similar topics and all included papers.

**Eligibility criteria for selecting studies:**

(1) Primary research published in a peer-reviewed journal; (2) inclusion of participants with no history of CVD; (3) intervention strategy consisted of provision of a CVD risk model estimate to either professionals or patients; and (4) the only difference between the intervention group and control group (or the only intervention in the case of before-after studies) was the provision of a CVD risk model estimate.

**Results:**

After duplicates were removed, the initial electronic search identified 9671 papers. We screened 196 papers at title and abstract level and included 17 studies. The heterogeneity of the studies limited the analysis, but together they showed that provision of risk information to patients improved the accuracy of risk perception without decreasing quality of life or increasing anxiety, but had little effect on lifestyle. Providing risk information to physicians increased prescribing of lipid-lowering and blood pressure medication, with greatest effects in those with CVD risk >20% (relative risk for change in prescribing 2.13 (1.02 to 4.63) and 2.38 (1.11 to 5.10) respectively). Overall, there was a trend towards reductions in cholesterol and blood pressure and a statistically significant reduction in modelled CVD risk (−0.39% (−0.71 to −0.07)) after, on average, 12 months.

**Conclusions:**

There seems evidence that providing CVD risk model estimates to professionals and patients improves perceived CVD risk and medical prescribing, with little evidence of harm on psychological well-being.

**Strengths and limitations of this study**

- This systematic review is the first to address the impact of provision of cardiovascular disease risk model estimates on patients’ or physicians’ behaviour or health outcomes.
- The use of broad inclusion criteria and the systematic search of multiple databases allowed us to include studies in which assessment of the impact of provision of a risk score alone was not the primary outcome.
- Despite this, the small number and heterogeneity of included studies limit the strength of conclusions that can be made. Most report only short-term changes and those that address behaviour change use mostly self-reported measures and are underpowered to detect small changes that may be clinically important at the population level.

## Background

Even though there have been advances in diagnosis, treatment and prevention of cardiovascular disease (CVD) in recent decades, CVD still remains the single largest cause of death worldwide.^1^ In 2011, 3 in every 10 deaths were caused by CVD,^2^ and it is estimated that by 2030, 23.3 million people will die annually due to CVD.^3^ This has led to increasing focus on affordable effective preventive strategies. These include collective approaches targeting the wider underlying determinants of CVD in an attempt to shift the entire population distribution of CVD risk factors, and approaches that focus on identification of individuals at high risk. An integral part of the latter approach is the use of CVD prognostic models or risk scores, such as the Framingham risk score,^4^ QRISK,^5^ ASSIGN,^6^ SCORE,^7^ PROCAM^8^ and Reynolds^9^ which share a core set of established risk factors (age, sex, smoking, blood pressure and total cholesterol) among other risk factors (eg, Townsend score, family history). These scores enable estimation of an individual's risk of developing CVD, and so have the potential to help physicians with decisions regarding initiation, type and intensity of treatment (eg, cholesterol-lowering treatment and blood pressure management), to facilitate an informed discussion between physician and patient regarding lifestyle changes and pharmacological treatment, to improve risk perception of both physicians and patients, and to motivate individuals to improve their health-related behaviours, with the ultimate goal to prevent CVD events. They also provide an opportunity to prioritise individuals with the highest CVD risk and so allocate resources efficiently.

Such risk models have been incorporated into many major clinical guidelines for routine practice^10–14^ and the UK National Health Service (NHS) Health Checks programme which aims to assess CVD risk for all those aged 40–74 years without pre-existing CVD. Despite this strong advocacy of the use of such CVD risk models, relatively little is known about the benefits and harms of provision of CVD risk model estimates to patients, and whether their use by physicians actually translates into improved behavioural and clinical outcomes. Previous groups have reviewed randomised clinical trials of the effectiveness of healthcare professionals using a CVD risk model or score to aid primary prevention,^15^ the effectiveness of the use of CVD risk model when combined with lifestyle interventions in the prevention of CVD,^16^ the effects of providing individuals with global CVD risk information with or without tailored interventions,^17^ and the effects of providing CVD risk model estimates on physician knowledge of global CHD risk.^18^ These systematic reviews all included studies in which the provision of a risk model estimate was part of a multifactorial intervention. To our knowledge, no recent systematic review has comprehensively addressed the specific impact of provision of a CVD risk model estimate to either practitioners or patients.

The purpose of this review was, therefore, to assess whether provision of a CVD risk model estimate to either patients or practitioners, as opposed to other simultaneous or subsequent interventions, such as lifestyle advice or exercise programmes, impacts patient or practitioner behaviour or health outcomes.

## Methods

We performed a systematic literature review following an a priori established study protocol (available on request). Reporting was according to the PRISMA statement.^19^

### Search strategy

As part of a larger systematic review on CVD risk scores, we performed an electronic literature search of MEDLINE and PubMed from 01/01/2004 to 01/06/2013 with no language restriction. The search strategy is described in full in online supplementary appendix 1. Briefly, it included terms for CVD, coronary heart disease, hypertension, hyperlipidaemia, stroke or cerebrovascular disease in combination with terms for risk assessment, prediction, score or decision support, and named risk scores. We also reviewed the reference lists of systematic reviews on this topic^15–18^ for studies published prior to 2004 and manually screened the reference lists of all included papers.

### Study selection

We included studies that met the following criteria: (1) primary (randomised and non-randomised) studies published in a peer-reviewed journal; (2) inclusion of participants with no history of CVD; (3) intervention strategy consisted of provision of a CVD risk model estimate to either physicians or patients (ie, not just providing a means by which physicians or patients could calculate CVD risk score); and (4) the only difference between the intervention group and control group (or the only intervention in the case of before-after studies) was the provision of a CVD risk model estimate. Observational and qualitative studies, studies calculating CVD scores for the secondary prevention of CVD or including both primary and secondary prevention where it was not possible to separate out the primary prevention group, and conference abstracts, editorials, commentaries, letters and reviews were excluded.

We selected studies in a three-stage process. In the first stage, titles of all studies identified from the electronic search were screened in duplicate by six reviewers involved in a large systematic review on CVD risk prediction led by ES and KGMM to identify all studies that described the application of a risk model into clinical practice or focused on risk-based management. In the second stage, this process was repeated with seven reviewers based on abstract. In the third stage, we combined those studies identified from stage 2 with studies from systematic reviews on similar topics,^15–18^ and two researchers (JU-S+SG/BS) independently screened the titles and abstracts against the inclusion and exclusion criteria. For studies where a definite decision to reject could not be made based on title and abstract alone, we obtained the full paper for detailed assessment. Two reviewers (JU-S and BS) then independently assessed the full-text articles for the possibility of inclusion in the review. We excluded papers identified by both researchers as not meeting the inclusion criteria. Any disagreements were resolved by discussion, and a final decision was made at consensus meetings by JU-S, BS and SG.

### Data extraction

JU-S and BS independently extracted data from all studies included in the review using a standardised data extraction form to reduce bias. The data extracted included: (1) study characteristics (research question, risk model or score used, study design, study setting, intervention, duration of follow-up, outcomes measured); (2) selection of participants (inclusion criteria, method of recruitment/randomisation); (3) participant characteristics (sample size, age, gender, comorbidity, level of CVD risk); and (4) measured outcome(s). Reviewers were not blinded to publication details. We requested additional unpublished data from the authors of papers in which it was mentioned that additional data were available or additional data were required to meet the inclusion criteria or for clarification of results.

### Quality assessment

JU-S and BS conducted quality assessment at the same time as data extraction. Since our review included studies with different designs, we used a checklist based on the Critical Appraisal Skills Programme guidelines for cohort studies and randomised controlled trials (RCTs; available from http://www.casp-uk.net/#!casp-tools-checklists/c18f8) as an initial framework and then classified each study as high, medium or low quality. No studies were excluded based on quality assessment alone.

### Data synthesis and statistical analysis

For analysis, we grouped the measured outcomes into those relating to risk perception, changes in health-related behaviour, intermediate outcomes (eg, blood pressure, cholesterol levels), modelled cardiovascular risk, medication prescribing, anxiety and psychological well-being, and contact with healthcare professionals after provision of risk information. For data on continuous outcomes, where possible, we expressed results as the difference in the mean change between groups. Where standardised mean changes were presented in the studies, we used the SD of the control group to convert data to non-standardised changes. Where this was not possible, we presented the results as mean±SD. For data on binary variables, such as a change in prescribing or meeting targets, we presented data as ORs or relative risk and 95% CIs. Where possible we combined data from different studies using random effects meta-analysis, but due to variations in study design and reporting, we were only able to do this for a small number of outcomes. We analysed all data according to the different outcomes and the recipient of the CVD risk score (physician or patient). For outcomes with data from three or more studies, we assessed the heterogeneity between studies using the I^2^ statistic. We did not perform formal tests of heterogeneity for outcomes with data from less than three studies. All analyses were conducted using statistical software package STATA/SE V.12. Significance was set at p<0.05.

## Results

**Figure 1:**
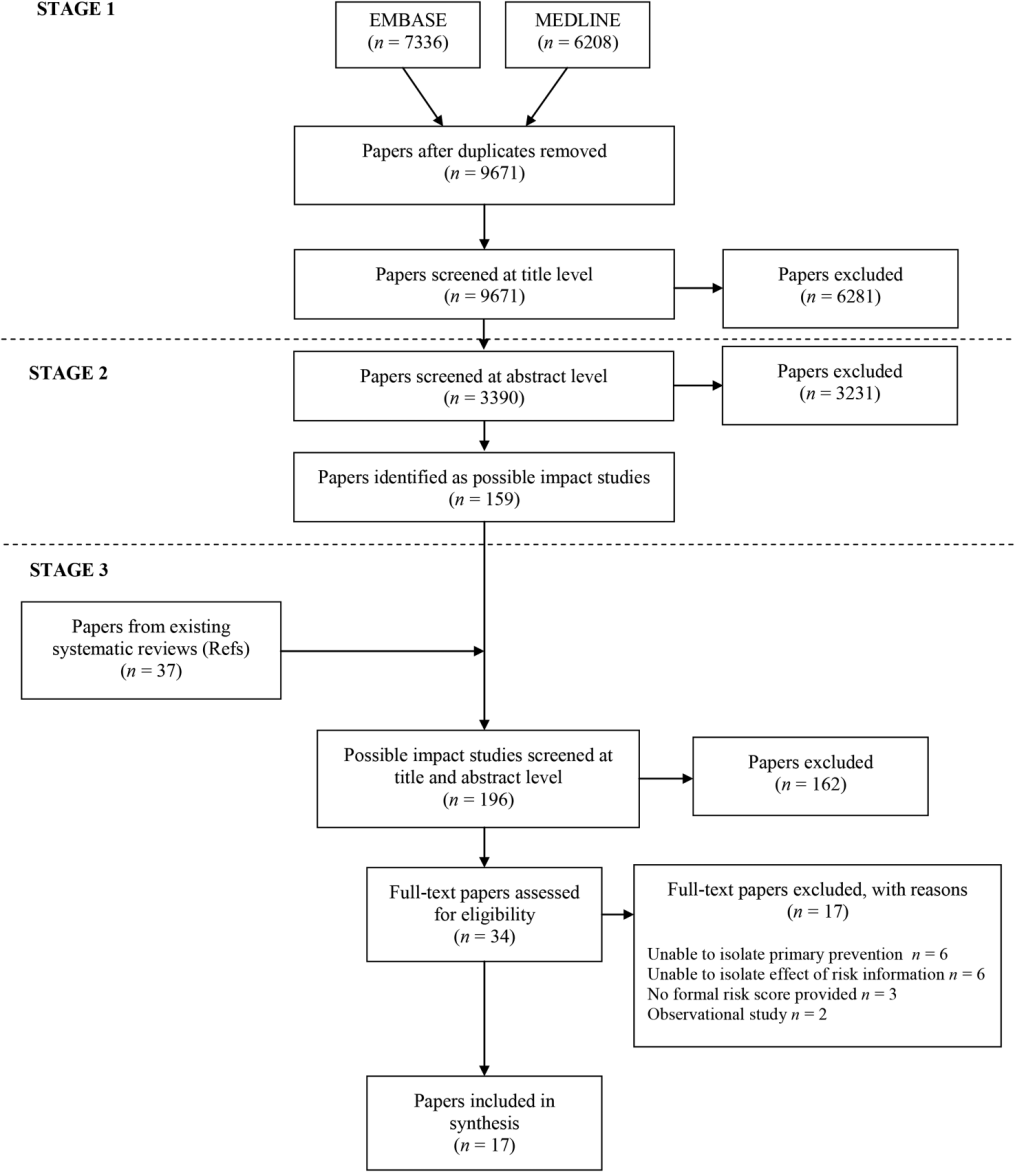
PRISMA flow diagram.

After duplicates were removed, the initial electronic search identified 9671 papers (figure 1). One hundred and fifty-nine of these were identified as possible inclusion papers during stage 1 and 2 of the screening. When the papers from existing systematic reviews were added to this number, we screened 196 papers at title and abstract level against the inclusion criteria. We excluded 162 and a further 17 after full-text assessment. The most common reasons for exclusion were that we were unable to isolate data on primary prevention or the effect of giving risk information alone (figure 1). The analysis is, therefore, based on 17 studies.^20–36^

A summary of the characteristics of those 17 studies is shown in table 1. They showed considerable heterogeneity in terms of size, setting, risk score used, duration of follow-up and outcomes measured. Seven (2 RCTs and 5 before-and-after studies) measured changes in understanding or risk, risk perception, psychological well-being or anxiety with 10 (9 RCTs and 1 before-and-after study) reporting changes in risk factors, prescribing or calculated risk. Eleven provided risk information to patients alone, three to physicians and three to both patients and physicians. Study quality assessment is summarised in table 2. Overall, it was variable with only one study being judged as high quality. All nine RCTs reporting changes in risk factors, prescribing or calculated risk were judged as medium or high quality. The effect sizes between the different studies are, therefore, more likely to reflect between-study heterogeneity for a number of study characteristics, in particular differences in the patient populations and delivery of risk information, than overall quality of the studies.

**Table 1:** Characteristics of studies

Author and date	Study design	Country	Recipient of risk information	Control group	Intervention group	Risk score provided	Duration of follow up	Outcomes measured	Quality assessment*
Asimakopoulou (2008)^20^	Before-after study	England	Patient	NA	Calculation of CVD/stroke risk followed by explanation of risk and discussion about difference between patients’ perception and actual risk	1, 5 or 10 year UKPDS V.2.0	6 weeks	Understanding and recall of risk	L
Avis (1989)^21^	RCT	USA	Patient	Baseline interview and assessment of perceived risk then follow-up interview at 7–12 weeks	Baseline interview, assessment of perceived risk and then health risk appraisal using one of four risk instruments and feedback on risk then follow-up interview at 7–12 weeks	CDC/HRA;^37^ The Heart Test;^38^ RISKO;^39^ Determine Your Medical Age^40^	7–12 weeks	Change in perceived risk	L
Christensen (1995)^22^	Before-after study	Denmark	Patient	NA	Health examination with calculation of risk score and health talk with the GP	Risk of coronary artery disease^41^	6 months	Change in psychological well-being	L
Christensen (2004)^23^	RCT	Denmark	Patient	Baseline questionnaire	Baseline questionnaire plus health screening with written feedback from their GPs and either optional or planned health discussions with their GP (2 intervention groups)	Risk of cardiovascular disease (modified from^41^)	1 and 5 years	Change in psychological well-being	L-M
Connelly (1998)^24^	Before-after study	UK	Patient	NA	Baseline questionnaire and screening appointment with provision of risk score. Participants at high risk were offered an appointment with a nurse or GP to discuss in more detail	5-year risk of CHD based on Northwick Park Heart Study^42^	10 days and 3 months	Change in psychological well-being and anxiety	M-H
Hanlon (1995)^25^	RCT	Scotland	Patient	Health education (interview backed up by written information) or health education and feedback on serum cholesterol	Health education plus feedback on risk score or health education and feedback on serum cholesterol plus feedback on risk score	Dundee risk score^43^	5 months	Self-reported change in diet, alcohol and smoking cessation, reduction in plasma cholesterol, and reduction in risk score	M
Hussein (2008)^26^	Before-after study	USA	Patient	NA	Provision of 5-year CVD risk estimate in interview lasting approximately 5 min	5-year Framingham risk	Immediate	Accuracy of risk perception	M
Paterson (2002)^27^	Before-after study	Canada	Patient	NA	A consultation lasting approximately 18 min with a GP working through a workbook covering CHD and the concepts of risk and the patient's absolute and relative risk	10-year risk of a coronary event based on Framingham Heart Study^44^	Mean 12.8±13.1 days	Change in perceived risk	L
Persell (2013)^28^	RCT	USA	Patient	Usual care	Patients were mailed a risk message containing their personal CVD risk information and encouraging them to discuss risk-lowering options with their primary care physician	10-year Framingham risk score	9 and 18 months	LDL cholesterol, BP, prescriptions for lipid-lowering or antihypertensive medication, smoking cessation and number of primary care physician contacts	M
Price (2011)^29^	RCT	UK	Patient	Told their individual fasting glucose level, blood pressure and LDL cholesterol and whether they were elevated according to current guidelines±brief lifestyle advice intervention	A 10-year cardiovascular risk estimate for current risk and ‘achievable risk’ calculated assuming current targets for systolic BP, LDL cholesterol, HbA1c and smoking cessation were met±brief lifestyle advice intervention	10 year UKPDS V.3.0 risk of cardiovascular disease	1 month	Physical activity, 10-year CVD risk, weight, body fat percentage, BP, alcohol consumption, LDL, triglycerides, fructosamine, fasting glucose, 2 h glucose, vitamin C, cotinine, anxiety, quality of life, self-regulation, worry about future risk of heart attack, intention to increase physical activity and prescribing	M-H
Qureshi (2012)^30^	Before-after study	UK	Patient	NA	Cardiovascular risk assessment then risk score along with lifestyle advice leaflet posted within 4 weeks. Participants with risk >20% offered appointment with their family physician or nurse 2 weeks later	10-year JBS2 cardiovascular risk score	6 months	Anxiety score, self-reported fat and unsaturated fat intake, smoking status and stage of change for increasing exercise	M
Bucher (2010)^31^	RCT	Switzerland	Physician	Physicians received booklet of evidence-based guidelines for the management of CHD risk factors and were advised in the booklet to access a website for CHD risk assessment	Physicians received same booklet of evidence-based guidelines plus a risk profile for each patient on the patient charts	10-year Framingham risk	12–18 months	Change in total cholesterol, blood pressure, Framingham risk score and initiation of medication	H
Hall (2003)^32^	RCT	Scotland	Physician	Usual care—physicians were unaware of ongoing study	Documentation of New Zealand Cardiovascular score at the front of medical records	5-year cardiovascular risk from New Zealand Cardiovascular score^45^	Not given	Change in prescribing for diabetes, hypertension or lipid-lowering drugs	M
Hanon (2000)^33^	RCT	France	Physician	Baseline measurement of BP and prescription of fosinopril followed by visits at 4 and 8 weeks at which physicians could add in hydrochlorothiazide	As for control group plus calculation of Framingham risk also given to physicians	10 year Framingham risk	8 weeks	Change in blood pressure, number of patients with dual antihypertensive therapy and change in Framingham risk	M
Grover (2007)^34^	RCT	Canada	Physician and patient	Physicians attended full-day educational session. Patients received usual care with follow-up at 2–4 weeks and 3,6,9 and 12 months	Physicians attended the same full-day educational session. Patients were given a copy of their risk profile and then followed up at 2–4 weeks, 3,6,9 and 12 months	10-year Framingham risk	12 months	Change in 10-year risk of CVD and probability of reaching lipid targets	M-H
Grover (2009)^35^	RCT	Canada	Physician and patient	Physicians attended full-day educational session. Patients received usual care with follow-up at 2–4 weeks and 3,6,9 and 12 months	Physicians attended the same full-day educational session. Patients were given a copy of their risk profile and then followed up at 2–4 weeks, 3,6,9 and 12 months	10-year Framingham risk	12 months	Mean blood pressure threshold for intensifying antihypertensive treatment	M
Lowensteyn (1998)^36^	RCT	Canada	Physician and patient	Physicians—1 h education meeting and a monthly newsletter. Patients—completed questionnaire about attitudes and knowledge surrounding CVD prevention and assessment of their current lifestyle and medical problems	Physicians—same 1 h education meeting and a monthly newsletter plus received 2 copies of patients risk profile within 10 working days. Patients—completed same questionnaire and then invited back 2 weeks later when presented with risk	8-year coronary risk from CHD Prevention Model and estimated ‘cardiovascular age’	3–6 months	Patient/physician follow-up decisions and changes in smoking, cholesterol, BP, BMI, 8-year coronary risk and cardiovascular age	L

*Low (L), medium (M), high (H).

BMI, body mass index; BP, blood pressure; CHD, coronary heart disease; CVD, cardiovascular disease; GP, general practitioner; HbA1c, glycated haemoglobin; LDL, low-density lipoprotein; NA, not available; RCT, randomised controlled trial.

**Table 2:** Quality assessment based on the Critical Appraisal Skills Programme guidelines

Author and date	Addressed a clearly focused issue	Appropriate method	Recruitment and comparability of study groups	Blinding	Exposure measurement	Outcome measurement	Follow-up	Confounding factors	Analysis	Results	Overall
Asimakopoulou (2008)^20^	•	•	•	•	•	•	•	•	•	•	L
Avis (1989)^21^	•	•	•	•	•	•	•	_	•	•	L
Christensen (1995)^22^	•	•	•	_	•	•	•	•	•	•	L
Christensen (2004)^23^	•	•	•	•	•	•	•	_	•	•	L-M
Connelly (1998)^24^	•	•	•	•	•	•	•	•	•	•	M-H
Hanlon (1995)^25^	•	•	•	•	•	•	•	_	•	•	M
Hussein (2008)^26^	•	•	•	_	•	•	•	•	•	•	M
Paterson (2002)^27^	•	•	•	_	_	•	•	•	•	•	L
Persell (2013)^28^	•	•	•	•	•	•	•	_	•	•	M
Price (2011)^29^	•	•	•	•	•	•	•	_	•	•	M-H
Qureshi (2012)^30^	•	•	•	•	•	•	•	•	•	•	M
Bucher (2010)^31^	•	•	•	•	•	•	•	_	•	•	H
Hall (2003)^32^	•	•	•	•	•	•	•		•	•	M
Hanon (2000)^33^	•	•	•	•	•	•	•	_	•	•	M
Grover (2007)^34^	•	•	•	•	•	•	•	_	•	•	M-H
Grover (2009)^35^	•	•	•	•	•	•	•	_	•	•	M
Lowensteyn (1998)^36^	•	•	•	•	•	•	•	_	•	•	M

L, low; M, medium; H, high.

Table 3 shows additional details about the participants in each of the studies, including the inclusion criteria, methods of recruitment and randomisation, and the baseline CVD risk of participants. Of the nine RCTs reporting changes in risk factors, prescribing or calculated risk, five included only high-risk patients (CVD risk estimate ≥20%) or those with uncontrolled hypertension or untreated hyperlipidaemia.^28^ ^29^ ^33–35^ The other four^25^ ^31^ ^32^ ^36^ included both high-risk and low-risk participants, with the proportion of each well balanced between the intervention and control groups.

**Table 3:** Details of participants

Author and date	Study design	Inclusion criteria	Method of recruitment/randomisation	n (% of those eligible)	Age (years)	Gender (male)	Baseline CVD risk score
Asimakopoulou (2008)^20^	Before-after study	Patients with type 2 diabetes free from cardiovascular, cerebrovascular or psychiatric comorbidity and able to understand English	Inspection of medical records then letter of invitation and randomisation to 1,5 or 10-year risk	95 (66%)	Mean 64 (range 42–72)	44%	Mean CHD 25% Mean stroke 15%
Avis (1989)^21^	RCT	Adults 25–65 years with no history of CHD, diabetes or hypertension	Random digit dialling then randomisation to one of 4 risk appraisal tools	Control: 89 Intervention: 542	NA	NA	Above average risk (risk ratio over 1.25): 36%
Christensen (1995)^22^	Before-after study	40–49-year-old men	Randomly selected from Public Health Insurance register then invited by GP	Low / moderate risk: 150 (81%) High risk 123 (73%)	40–49	100%	NA
Christensen (2004)^23^	RCT	30–49 years old registered with local GP	Letter of invitation to random sample of those registered with local practice then randomisation into control and 2 intervention groups (combined for analysis)	Control: 501 (75%) Intervention: 905 (68%)	NA	NA	High risk (score>10): 11.4%
Connelly (1998)^24^	Before-after study	Men aged 45–69 years with no obvious contraindications to antithrombotic therapy, no history of peptic ulceration or previous history of MI, stroke or serious psychiatric disorder	Search of medication records then letters of invitation	Baseline: 5772 (99%) 10 days: 4917 (85%) 3 months: 4244 (74%)	45–69	100%	High risk (highest quintile): 18.4%
Hanlon (1995)^25^	RCT	Workers at two work sites not working permanent night shifts, taking part in another coronary intervention or taking lipid-lowering medication	Random selection of workers then computer-generated randomisation	Control: 229 (78%) (HE only) and 226 (76%) (HE and feedback on cholesterol) Intervention: 214 (75%) (HE and risk) and 199 (76%) (HE, feedback on cholesterol and risk)	Control: 20–65 Intervention: 20–65	NA	NA
Hussein (2008)^26^	Before-after study	People with complete data available and no history of CVD events	Self-selection at 2 events of free stroke risk screening as part of a community health fair	146 (80%)	Mean 47±15	36%	High risk (5%): 23.97%
Paterson (2002)^27^	Before-after study	Age 30–74 years with measurements for blood pressure, smoking status, total and high-density lipoprotein cholesterol and free from cardiovascular disease	First 20 physicians who responded to letter of invitation. Physicians then enrolled 2 patients who met the study criteria and for whom they would be likely to use Heartcheck under normal practice conditions	37 (92.5%)	50±10.7	68%	Mean (SD): 10.8% (6.9)
Persell (2013)^28^	RCT	Primary care physicians at an academic medical centre and patients aged 40–79 years without history of CVD, DM or PAD, not taking lipid-lowering medication who had 2 or more clinic visits in the preceding 2 years and LDL cholesterol test in previous 5 years with most recent LDL ≥100 mg/dL and 10-year FRS >20% or LDL ≥130 mg/dL and FRS 10–20% or LDL ≥160 mg/dL and FRS 5–10%	Medical record search then block randomisation at the level of the practice using random number generator	Control: 217 Intervention: 218 (93.6% across both control and intervention groups)	Control: 60.1±9.2 Intervention: 61.3±9.4	Control: 77% Intervention: 77.5%	Mean (SD): 14% (6.5)
Price (2011)^29^	RCT	Patients with CVD risk ≥20%, able to read and write English, not known to have CVD or a physical disability or other condition reducing the ability to walk	Eligible patients mailed written invitation and then factorial computerised randomisation	Control: 91 Intervention: 94 (16% across both control and intervention groups)	Control: median (IQR) 62.4 (56.0–65.9); Intervention: median (IQR) 62.3 (54.2–66.2)	Control: 71% Intervention: 64%	Median (IQR) men: 48% (34–60); women 31% (22–43)
Qureshi (2012)^30^	Before-after study	Aged 30–65 years requesting a CVD risk assessment by their family physician without previous diagnosis of diabetes or CHD, stroke or PAD and not already receiving lipid-lowering medications or excluded by their physicians for psychological or social reasons	Usual practice	Control: 353 (92.9%) Intervention: 305 (80.3%)	Median 52 (45–58)	39%	High risk (>20%): 11.4%
Bucher (2010)^31^	RCT	Patients registered at the centres, not pregnant, aged 18 or older with continuous cART for 90 days prior to baseline and with complete data on CHD risk factors at baseline	Physicians randomised in strata according to patient volume and type of setting	Control physicians: 57 (71%) Control patients: 1682 (84%) Intervention physicians: 60 (71%) Intervention patients: 1634 (78%)	Control: median 44 (39–50) Intervention: median 44 (39–51)	Control: 64% Intervention: 68%	High risk (>20%): 3%
Hall (2003)^32^	(R)CT	Patients 35–75 years with type 2 diabetes and no history of CVD or renal disease attending a hospital outpatient clinic	Consecutive recruitment of patients with alternate allocation to experimental and control group with doctors unaware of project	Control: 161 Intervention: 162	NA	NA	High risk (>20%): 52%
Hanon (2000)^33^	RCT	Adults 18–75 years with BP >140/90 without severe hypertension, secondary hypertension, heart disease, CVD, renal, pulmonary, hepatic disease or significant psychiatric or other serious illness, diabetes, pregnancy or of reproductive age without effective contraception	Recruited during usual care then randomised into 2 groups whether primary care physician had been told CVD risk	Control: 712 Intervention: 556	Control: Mean 60±10 Intervention: Mean 60±10	Control: 54% Intervention: 54%	Mean (SD): 25.4% (12.0)
Grover (2007)^34^	RCT	Patients with diabetes or 10-year risk > 30% with moderate cholesterol, 10-year risk 20–30% with high cholesterol or 10-year risk 10–20% with very high cholesterol with no hypersensitivity to statins, risk of pregnancy, breastfeeding, active liver disease, raised CK or triglycerides, a history of pancreatitis or significant renal insufficiency*	Identified from office medical records or prebooked clinic appointments then randomisation stratified by risk level	Control: 1193 Intervention: 1163 (initial 98% recruitment)	NA	NA	Mean (SD): 17.8% (7.5)
Grover (2009)^35^	RCT	As for Grover 2007^34^	Identified from office medical records or pre-booked clinic appointments then randomisation stratified by risk level	Control: 143 Intervention: 166 (initial 98% recruitment)	NA	NA	Mean (SD): 17.8% (7.5)
Lowensteyn (1998)^36^	RCT	Interested primary care physicians around study centre. Patients 30–74 years without history of CVD in whom clinicians thoughts a risk profile would be clinically useful	Practices around study centre with block randomisation at the level of primary care practice according to presence or absence of medical school. Patients selected by physicians	Control physicians:32 (39%) Control patients: 176 Intervention physicians: 97 (57%) Intervention patients: 782	Control: 50.7±11.3 Intervention: 50.0±10.8	Control: 64.8% Intervention: 64.8%	Mean (SD): 10.5% (9.3)

*Full criteria for inclusion were: 10-year risk >30% with LDL ≥97 mg/dL or TC:HDL ratio ≥4 or; 10-year risk 20–30% with LDL ≥116 mg/dL or a TC:HDL ratio ≥5 or; 10-year risk 10–20% with HDL-C ≥155 mg/dL or TC:HDL-C ratio ≥6; no hypersensitivity to statins, risk of pregnancy, breast feeding, active liver disease or elevated AST or ALT levels (>3 times normal), CK ≥5 times normal, elevated TGs (>939 mg/dL), a history of pancreatitis or significant renal insufficiency.

ALT, alanine transaminase; AST, aspartate transaminase; cART, combination antiretroviral therapy; CHD, coronary heart disease; CK, creatine kinase; CVD, cardiovascular disease; DM, diabetes mellitus; FRS, Framingham risk score; GP, general practitioner; HDL, high density lipoprotein cholesterol; LDL, low-density lipoprotein cholesterol; MI, myocardial infarction; NA, not available; PAD, peripheral arterial disease; RCT, randomised controlled trial; TC, total cholesterol; TG, triglyceride.

### Risk perception

Two before-and-after studies reported the immediate effects of receiving risk information. In one, patients tended to initially overestimate their risk and giving risk information resulted in a significant reduction in perceived risk (eg, mean perceived 10-year risk of CVD fell from 48% to 20%, n=95, p<0.001).^20^ The other reported a significant increase in the proportion with accurate risk perception from 34% to 74% (p<0.0001) following risk information mostly due to a reduction in the rate of underestimation (60% to 18% (p<0.0001)), while the rate of overestimation was low and did not change (7% to 8%; p=0.82; n=146).^26^ However, Price *et al* showed in their RCT that the risk perception after 4 weeks did not differ between those who received the risk estimate only (mean 33.7, SD 18.9) and those who did not (mean 34.8, SD 19.6; p=0.87). Similar pattern of results were present when the comparison was made between those who received risk estimate + lifestyle advice (mean 35.6, SD 18.6) and those who received lifestyle advice only (mean 41.8, SD 20.7, p=0.14).

Three studies also reported changes in risk perception at different time intervals after receipt of risk information. In two, patients tended to initially overestimate their risk and giving risk information resulted in a sustained significant reduction in perceived risk after 2–4 weeks (mean 18% from 32%, n=37, p<0.00 001)^27^ and 6 weeks (eg, mean perceived 10-year risk of CVD was 26% after 6 weeks compared with 48% prior to risk information, n=95, p<0.001).^20^ Asimakopoulou *et al*^20^ additionally showed that, controlling for actual risk, those patients given their 10-year risk reported consistently higher risk estimates than those given 1-year or 5-year risk estimates, while the 1-year and 5-year groups’ estimates were not significantly different from each other. The third study reported change in perceived risk 7–12 weeks after provision of risk information in one of four formats (CDC, RISKO, Arizona Heart Institute or Medical age).^21^ Forty per cent were initially accurate (42% underestimating their risk and 18% overestimating risk) and the majority of respondents did not change their perceived risk. Those who received feedback that they were above average risk were more likely to increase their perceived risk, but all other groups were equally likely to increase their perceived risk as decrease with 10.4% of those who were told they were below average risk increasing their perceived risk and 11.9% of those who were told their risk was higher, decreasing their perceived risk.

### Changes in health-related behaviour

#### Diet

One before-and-after study in general practice^30^ and one RCT of factory workers^25^ included self-reported changes in diet. The provision of risk information led to no statistically significant changes in reported total fat intake or unsaturated fat intake at 6 months^30^ or the percentage who increased fruit and vegetable or fibre consumption or reduced fat at 5 months.^25^ In the latter study, although not statistically significant, the effects of provision of risk information to different groups ranged from a relative reduction of 10.6% to a relative increase of 47.4% in fibre intake. Only one study used an objective measure of changes in diet (plasma vitamin C) and also showed no significant effect of risk information among those with CVD risk >20% (standardised difference in means −0.079 (−0.37 to 0.21), p=0.589) at 1 month.^29^

#### Smoking cessation

Four studies reported on smoking cessation.^25^ ^29^ ^30^ ^36^ All showed no significant effect of risk communication irrespective of differences in study design (before-and-after study or RCT), duration of follow-up (1 to 12 months) or outcome measure (self-report or objectively measured cotinine): one found no significant difference in the percentage of smokers at 3 months with the mean difference adjusted for baseline and patients nested within the same physician (−0.8%, p=0.64, control n=89, intervention n=202);^36^ another measured smoking status among participants with CVD risk >20% using cotinine and also found no significant difference (standardised difference in means −0.53 (−1.23 to 0.17), p=0.136) at 1 month;^29^ the third, a before-and-after study, showed a small but non-significant increase in smoking (10.0% to 11.4% prerisk and postrisk information);^30^ and the fourth showed mixed results with more factory workers stopping smoking at 5 months in the group receiving risk information and health education than health education alone (9.6% vs 6.0%) but less stopping smoking in the group receiving risk information and feedback on cholesterol and health education than those receiving feedback on cholesterol and health education alone (3.6% vs 8.2%).^25^

#### Physical activity

Two studies reported on intention to increase physical activity. Both showed no effect with one before-and-after study including 11.4% of participants with CVD risk >20% showing no change in the number of participants in the action or maintenance stages of change for exercise (n=145/298 prerisk information and n=151/298 postrisk information)^30^ and the other RCT showing no significant between-group differences in intention to increase physical activity (no data given).^29^ Only one study assessed change in physical activity and reported no significant difference in mean accelerometer counts at 1 month among participants with CVD risk >20% (standardised difference in means 0.086 (−0.20 to 0.37), p=0.559).^29^

#### Alcohol consumption

Two RCTs showed no difference in self-reported alcohol consumption. One including only participants with CVD risk >20% reported no difference in mean alcohol intake at 1 month (standardised difference in means=−0.033 (−0.36 to 0.29), p=0.84)^29^ and the other no change in the proportion of factory workers who decreased alcohol consumption after 5 months among those drinking more than 21 units for men and 14 units for women at baseline (p=0.064).^25^

### Changes in intermediate measures

#### Cholesterol

**Figure 2:**
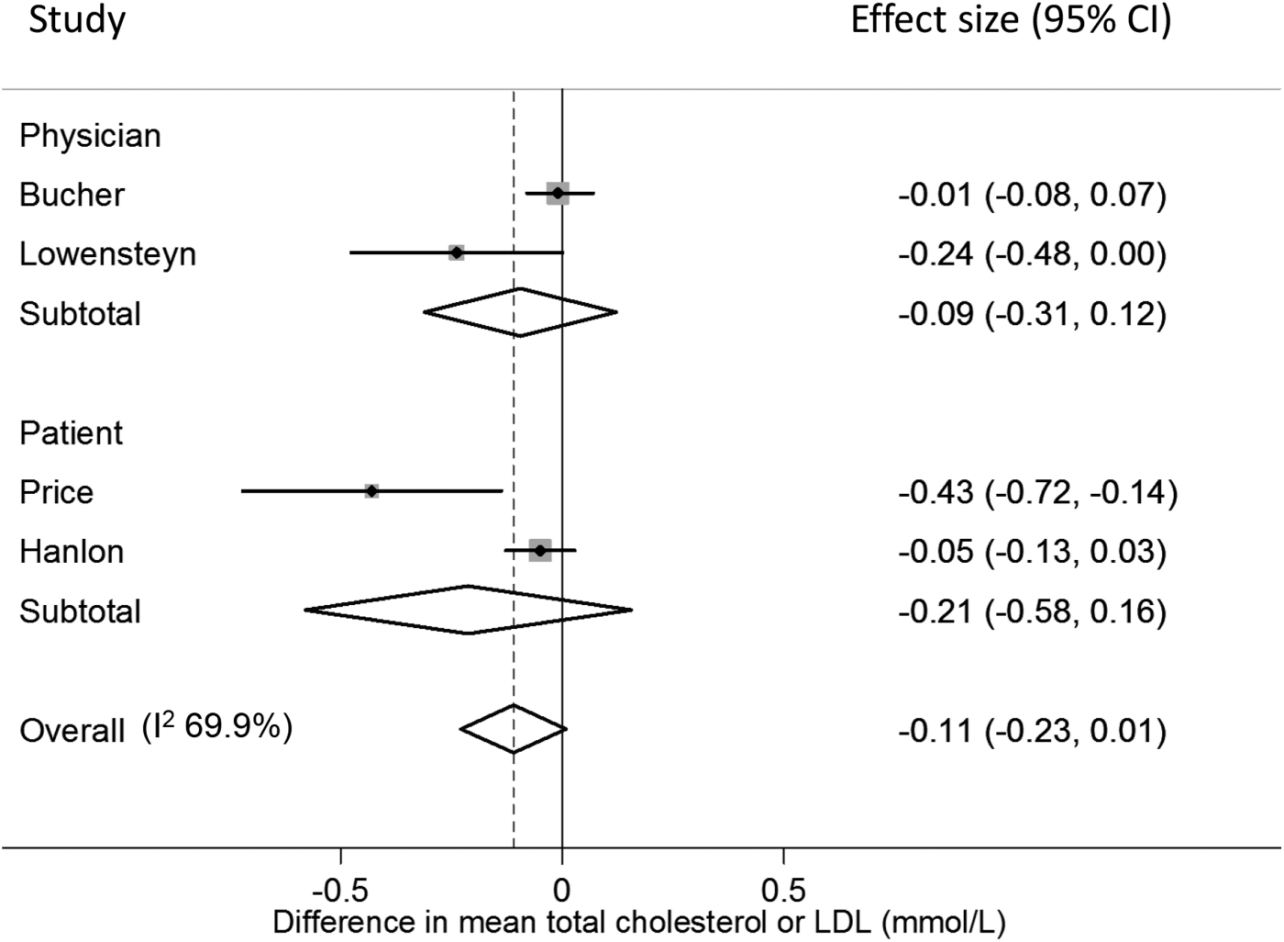
Forest plot showing the effect of provision of cardiovascular disease risk estimates to physicians or patients on the mean total cholesterol or low-density lipoprotein (LDL).

Four RCTs reported the between-group difference in mean change in total cholesterol or low-density lipoprotein cholesterol (LDL). Two were cluster randomised studies in which physicians in the intervention group received risk information about their patients.^31^ ^36^ After adjusting for lipid-lowering or antihypertensive medication at baseline^31^ and baseline characteristics and patients nested within the same physician,^36^ neither showed a difference in cholesterol after 3 months (figure 2).

In the two other trials, risk information was given to participants. In one, factory workers were randomised to receive either health education±risk information or health education and feedback on cholesterol±risk information. There was no significant difference in mean change in plasma cholesterol at 5 months (pooled difference in mean change −0.05 mmol/L (−0.13 to 0.03, p=0.208)) or 12 months (pooled difference in mean change −0.025 mmol/L (−0.103 to 0.025, p=0.529)).^25^ The other trial was the only one to include participants with CVD risk >20% and showed a significant difference in mean change in LDL after 1 month^29^ with sensitivity analysis excluding participants who had a change in drug treatment also showing a net 7.8% reduction in LDL (p<0.001).^29^ Combining the data for 5 months from Hanlon *et al* with Price *et al* gave a non-significant decrease, and pooling both with the other two studies showed a trend towards lower cholesterol but this was not significant (p=0.069, I^2^=69.9%; figure 2).

No significant differences were found with high-density lipoprotein cholesterol (HDL) or total cholesterol to HDL ratio after adjusting for baseline risk,^36^ or with triglycerides^29^ or the number of patients who had had their LDL repeated and in whom it was 30 mg/dL or lower than baseline at 9 months (OR 0.99 (0.56 to 1.74))^28^ among those with CVD risk >20% or with raised LDL. One RCT of participants with untreated hyperlipidaemia did, however, find that patients were more likely to reach lipid targets if they received risk information (OR 1.26 (1.04 to 1.53), n=1163 (intervention) and 1193 (control)) and there was a significant interaction (p=0.04) between being given a risk profile and the age gap (estimated cardiovascular risk age minus actual age) with the OR for reaching lipid targets in individuals who were reassured that they were at low risk 0.92 (0.64 to 1.31) compared with 1.69 (1.21 to 2.36) for those in the highest age gap quintile.^34^

#### Blood pressure

**Figure 3:**
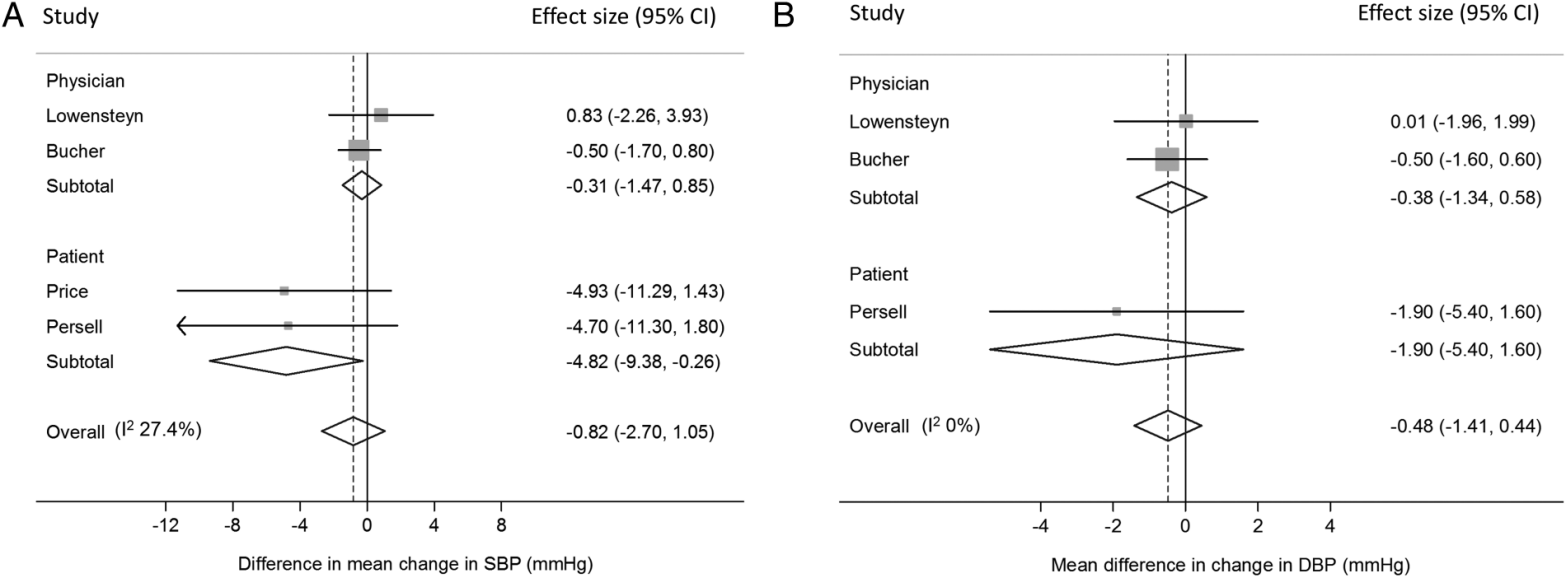
Forest plots showing the effect of provision of cardiovascular disease risk estimates to physicians or patients on the mean change in (A) systolic blood pressure (SBP) and (B) diastolic blood pressure (DBP).

Five RCTs reported the difference in mean change in blood pressure between patients with and without risk information.^28^ ^29^ ^31^ ^33^ ^36^ Three provided risk information to physicians.^31^ ^33^ ^36^ All three showed a non-significant difference in mean change in systolic blood pressure (SBP) or diastolic blood pressure (DBP). Pooling the data from Bucher *et al*^31^ which is adjusted for lipid-lowering medication or antihypertensive medication and Lowensteyn *et al*^36^ adjusted for group differences at baseline and patients nested within the same physician gave a non-significant reduction in both SBP and DBP (figure 3A,B). It was not possible to pool the data from Hanon *et al*^33^ because they reported only the pooled blood pressure preintervention and postintervention (intervention group (n=556); preintervention SBP 167, SD 13; and postintervention SBP 140, SD 11; and control group (n=712) SBP 166, SD 12, and 140, SD 10 preintervention and postintervention, respectively).

In the two other trials, risk information was provided to patients.^28^ ^29^ Both included only participants with CVD risk >20% or raised LDL, and together they showed a significant reduction in SBP in those patients that received risk information after 1 and 9 months (figure 3A). Persell *et al*^28^ additionally showed no significant effect on DBP (figure 3B), and combining all five studies together gave non-significant differences in both SBP and DBP (figure 3A, B).

#### Weight/BMI

Two studies reported changes in weight and found no significant difference in mean weight (standardised difference in means 0.065 (−0.22 to 0.35), p=0.66) or body fat percentage (standardised difference in means 0.063 (−0.23 to 0.35), p=0.67) among participants with CVD risk >20% at 1 month^29^ or mean change in body mass index (BMI) at 3 months adjusted for baseline and patients nested within the same physician (mean difference 0.154, p=0.31).^36^

#### Glycaemia

Price *et al*^29^ reported no significant change in fructosamine (standardised difference in means 0.207 (−0.08–0.50), p=−0.159), fasting glucose (standardised difference in means −0.024 (−0.31 to 0.26), p=0.87) or 2 h glucose (standardised difference in means −0.022 (−0.31 to 0.27), p=0.88) among participants with CVD risk >20% at 1 month.

### Changes in modelled cardiovascular risk

**Figure 4:**
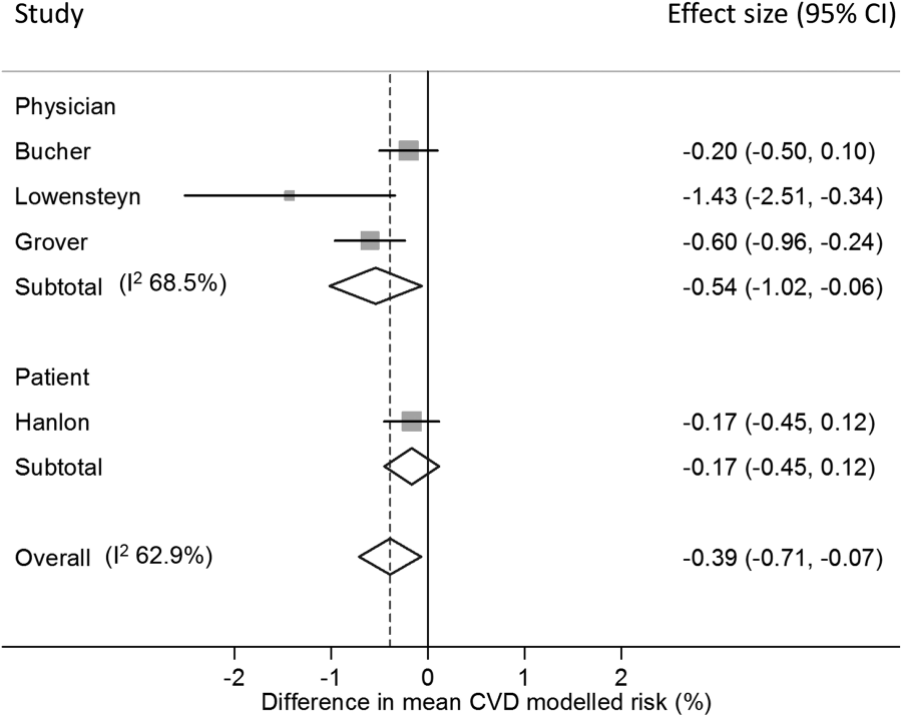
Forest plot showing the effect of provision of cardiovascular disease (CVD) risk estimates to physicians or patients on the mean change in modelled CVD risk.

Five RCTs reported changes in modelled CVD risk. In three, risk information was provided to physicians.^31^ ^34^ ^36^ Bucher *et al*^31^ reported difference in mean change in 10-year Framingham risk after 12–18 months in HIV patients after adjusting for lipid-lowering or antihypertensive medication; Lowensteyn *et al*^36^ reported the difference in mean change in 8-year coronary risk after 3–6 months adjusting for baseline and patients nested within the same physician; and Grover *et al*^34^ reported the difference in mean change in 10-year risk of CVD after 12 months adjusted for baseline. Together, they showed a statistically significant reduction in modelled risk (figure 4).

In the other two RCTs, risk information was provided to patients.^25^ ^29^ One found no significant difference in modelled risk at 1 month among those with CVD risk >20% (standardised difference in change in means −0.155±0.146, p=0.239).^29^ The other showed a non-significant increase at 5 months (difference in mean change −0.154 (−0.373 to 0.066), p=0.171 on a scale 1–100 where 1 is highest risk) and non-significant decrease at 12 months (difference in mean change 0.167 (−0.116 to 0.450), p=0.248) among factory workers.^25^ Combining the data from Hanlon *et al* at 12 months with the three studies providing risk information to physicians gave a significant reduction in risk score (figure 4).

### Changes in prescribing

#### Lipid-lowering medication

**Figure 5:**
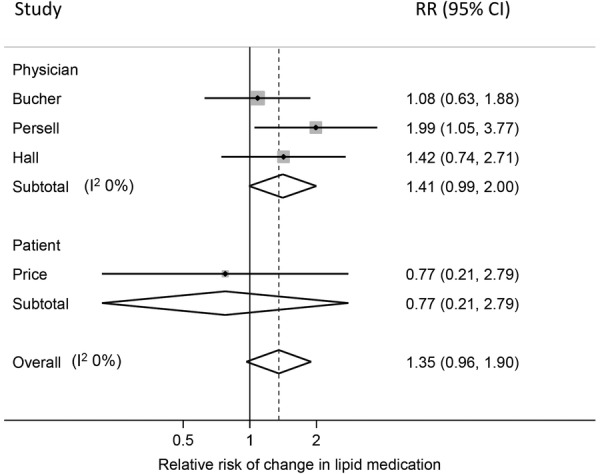
Forest plot showing the effect of provision of cardiovascular disease risk estimates to physicians or patients on the relative risk (RR) of receiving a change in lipid medication.

Four RCTs reported changes in lipid-lowering medication. One in which risk scores were provided to physicians blinded to the trial showed a 42% increase in the probability of having a change in lipid-lowering medication among all patients, but this was not statistically significant (p=0.29). In the same study, patients with a CVD risk >20% were twice as likely to have their medication changed when physicians were presented with the risk score (relative risk (RR) 2.32 (1.01 to 5.29), p=0.03).^32^ The other three trials reported the difference in the number of new prescriptions for lipid-lowering medication. Two gave risk information to physicians treating patients with HIV,^31^ or patients with CVD risk >20% or raised LDL,^28^ and one to patients with a CVD risk >20%.^29^ When pooled there was RR of 1.35 not achieving statistical significance (p=0.08, I^2^=0%; figure 5), but when only those studies including participants with CVD risk >20% or raised LDL are pooled, there is a significant increase in initiation of lipid medication (RR 1.83 (1.13 to 2.98)) which increases to 2.11 (1.27 to 3.49) when only the two studies providing risk information to physicians are included.

#### Blood pressure-lowering medication

Three RCTs reported changes in blood pressure-lowering medication. As with lipid-lowering medication, Hall *et al*^32^ showed no difference in the probability of a change in medication among all patients (RR 1.52 (0.86 to 2.69), p=0.146) but patients with a CVD risk >20% who received risk information were twice as likely to have their medication changed (RR 2.38 (1.11 to 5.10), p=0.0189). The other two included only participants with uncontrolled hypertension or CVD >20% or raised LDL and reported no difference in the percentage of patients requiring dual therapy after 8 weeks (46%, n=712 control vs 41%, n=556 risk information),^33^ the percentage of patients with uncontrolled hypertension at baseline who had an increase in number of antihypertensive drug classes (OR 2.89 (0.70 to 11.9) p=0.14)^28^ or the median number of antihypertensive drug classes used (p=0.45).^28^

One study including only participants whose blood pressure was above currently recommended targets reported the mean blood pressure threshold for intensifying treatment.^35^ They found no significant difference in the mean blood pressure thresholds for the whole study population (difference in means 2.8 mm Hg (−0.5 to 6.0) for systolic BP and 1.1 mm Hg (−0.9 to 3.2) for diastolic BP) or when stratified by age gap (the difference between their CVD risk age and chronological age).

#### Glucose-lowering medication

Three RCTs found no effect of risk information on either change in medication in all participants (RR 1.17 (0.89 to 1.53), p=0.273) or those with CVD risk >20% (RR 1.26 (0.87 to 1.84), p=0.219),^32^ or initiation of medication following provision of risk information to physicians caring for patients with HIV (RR 1.33 (0.30 to 5.96), p=0.704)^31^ or patients with CVD risk >20% (RR 6.78 (0.36 to 129.43)).^29^

### Psychological well-being and anxiety

Psychological well-being following receipt of risk information was assessed in four studies. Three showed no difference. In the first, a before-after study, there was no significant difference in the 12-item General Health Questionnaire (GHQ) score after 6 months in either those at low/moderate risk or those at high risk and no significant difference between the groups (n=146 in low/moderate group and 116 in high-risk group, p=0.80).^22^ A subsequent RCT also showed no significant difference in GHQ-12 after 1 year (p=0.603) or 5 years (p=0.727) between those receiving risk information (n=802, 745) and controls (n=390, 381).^23^ The third, also an RCT,^29^ measured change in general health after 1 month using the EQ-5D-3L and showed no between-group differences between those who received the risk estimate only (mean change 0.05, SD 0.12) and those who did not (mean change 0.01, SD 0.08; p=0.06). A similar pattern of results was seen when the comparison was made between those who received risk estimate + lifestyle advice (mean change −0.003, SD 0.08) and those who received lifestyle advice only (mean change 0.02, SD 0.11; p=0.442). The fourth used the GHQ-28 and showed a significant (p<0.005) reduction in mean scores from 7.21, SD 5.18 to 6.59, SD 5.7 in high-risk participants after 10 days.^24^

Three studies also explored changes in anxiety. Two were before-and-after studies and used the Spielberger state anxiety inventory. As with psychological well-being in the same study, a significant reduction in mean anxiety from 32.3, SD 8.72 to 28.1, SD 8.60 was seen in high-risk participants after 10 days (p<0.005, n=1676),^24^ but this was not seen in the other study including all participants after 6 months (median (IQR) prerisk 35 (26.7 to 43.3) and postrisk 33 (23.3 to 43.3)).^30^ The third, an RCT, reported no significant between-group difference in change of anxiety at 1 month measured by the six-item Spielberger state anxiety inventory when the comparison was made between those who received risk estimate only (mean change −0.45, SD 2.87) and control group (mean change −0.63, SD 2.61; p=0.707). A similar pattern of results was seen when the comparison was made between those who received risk estimate +lifestyle advice (mean change −0.27, SD 2.87) and those who received lifestyle advice only (mean change −0.64, SD 3.21; p=0.324). The same study also found no difference in worry about future risk of heart attacks, measured using the adapted Lerman breast cancer worry scale, or in self-regulation.^29^

### Contact with healthcare professionals

Two studies reported the effect on healthcare usage of giving risk information. In one,^36^ the design of the study encouraged control patients to see their physician in order to receive their risk score, and so follow-up was higher in the control group. There was, however, a significant difference between low-risk and high-risk individuals (p for interaction 0.026) with high-risk individuals being more likely to be followed up than low-risk individuals.

A further study in the USA including only participants with CVD risk >20% or with raised LDL showed no significant difference in healthcare usage at 9 months between those receiving risk information (n=218) and controls (n=217) with no difference in the percentage with any follow-up visit (p=0.28), number of follow-up visits (p=0.14), number of telephone (p=0.75) or email (p=0.96) contacts.^28^

## Discussion

### Principal findings

To our knowledge, this is the first systematic review to address specifically whether the provision of CVD risk model estimates alone impacts patient or practitioner behaviour or health outcomes. Despite the widespread adoption of risk scores in guidelines,^10–14^ only 17 studies were identified and they are heterogeneous in terms of size, design, baseline level of risk and choice of outcomes. They do, however, show that providing patients with risk information changes risk perception and increases the accuracy of perceived risk without decreasing quality of life or increasing anxiety. While there is no current evidence that this translates into changes in lifestyle, small reductions in cholesterol, blood pressure and modelled CVD risk are seen consistently. These may be mediated through changes in prescribing: providing risk information to physicians leads to statistically significant changes in prescribing of both lipid-lowering and blood pressure medications, with these effects greater in those at higher risk. However, trends towards small reductions in cholesterol and blood pressure were also seen in studies in which risk information was only provided to patients. Only one study included in our systematic review was judged as high quality and most of the studies relied on self-reported measures of health-related behaviour. Consequently, there is a need for further research with adequately powered trials and objective outcomes to better understand the impact on behaviour of provision of a CVD risk estimate to physicians and individuals.

### Strengths and weaknesses

The main strengths of this review are the use of broad inclusion criteria and the systematic search of multiple databases in addition to the inclusion of studies identified in previous systematic reviews on related topics. This allowed us to include studies in which assessment of the impact of provision of a risk score alone was not the primary outcome. While this reduces publication bias, the literature on CVD risk is diverse and rapidly expanding, and so it remains possible that there are additional studies of relevance that we were not able to identify. Furthermore, additional studies may have been published since June 2013, the date of the literature search. We are not aware, however, of any papers that would alter the overall conclusions of this review.

The other main limitation, as with most systematic reviews, is the extent and quality of the published data. Given the interest in CVD prevention and widespread use of CVD risk scores, it is both surprising and concerning that so few studies concerning their impact on care have been published. Additionally, of the 17 studies identified, most report only short-term changes (<6 months) and those that address behaviour change use mostly self-reported measures and are underpowered to detect small changes that may be clinically important at the population level. The small number and heterogeneity of the studies also made combining results difficult. Not only did they report different outcomes at different time periods with participants of varying baseline risk, but many of the studies adjusted for different baseline variables without reporting unadjusted changes or provided insufficient data to allow us to calculate effects adjusted for baseline risk. Data on the SD of outcomes were also not available for most studies, meaning it was not possible to calculate standardised changes in outcomes. A further limitation was that the small number of studies differed in whether the risk model estimates were presented to patients or physicians. This meant that in most cases there were only one or two studies presenting risk model estimates to the same group and reporting the same outcome. To provide the greatest interpretation of this limited data, where we were able to synthesise the data, in addition to presenting these separately, we also presented an overall summary estimate. Although this means these estimates need to be interpreted with caution, they probably reflect real life as in routine clinical practice this distinction is often not as clear cut: physicians will often discuss risk estimates with patients and patients will often ask physicians for advice on interpreting estimates.

### Implications for clinicians and policymakers

Despite these limitations, the results from this review are of relevance to the large number of clinicians worldwide who use CVD risk information with patients regularly in their practice, and policymakers involved in designing and implementing strategies for the prevention of CVD, including the recent debate about the NHS programme of CVD risk reduction in the UK.

The finding that providing patients with risk information changes risk perception and increases the accuracy of perceived risk is consistent with previous reviews which have shown that global risk information with accompanying education or counselling increases the accuracy of perceived risk.^17^ However, even immediately after being provided with risk information, 1 in 4 participants still had an inaccurate perceived risk^26^ and 1 in 10 changed their perceived risk in the opposite direction to the feedback they received.^21^ Such challenges to the communication of risk are well known,^46^ and there is scope for further work, but this highlights the need for clinicians to remain aware of the limitations of current methods.

With this inaccuracy in risk perception along with existing experience from other areas about the challenges of behaviour change, it is perhaps not surprising that there is no evidence that simply providing patients with a number leads to statistically significant changes in habitual environmentally cued behaviours such as diet, smoking, physical activity or alcohol intake. Where there were differences between the groups, most were in favour of providing risk information but the studies were generally of poor quality, underpowered and imprecise with most relying on self-reported information. While we are, therefore, unable to rule out the possibility of a small potentially clinically important effect, we can say that it appears unlikely that providing risk information will result in harm through false reassurance and the adoption of unhealthy behaviours. There was also either no change or an improvement in psychological well-being, anxiety, worry about future risk of heart attacks, or self-regulation in these studies. This is an important finding as screening programmes based on risk assessment such as the NHS Health Checks in the UK have the potential to cause harm and a key decision when considering implementation is the extent of that harm. Our review suggests that such screening programmes are safe when it comes to psychological well-being.

The effect of the provision of risk information on intermediate measures of CVD and modelled CVD risk was more consistent with studies consistently showing reductions in cholesterol, SBP and DBP, and modelled CVD risk. While the effect sizes are small, they may be of clinical significance at the population level and those studies including only high-risk participants showed significant reductions in cholesterol and SBP. It is possible these effects are mediated through changes in prescribing: although only on the border of statistical significance, patients were 1.4–1.5 times more likely to have a change of lipid-lowering or blood pressure medication when risk information was provided to their physicians and this increased to over twice as likely when only those with a CVD risk >20% were included. This difference in prescribing is not unexpected. Guidelines for prescribing lipid-lowering and blood pressure medication are based on assessment of CVD risk. The increased prescribing for those at high risk therefore likely reflects a greater number of physicians following existing guidance and the smaller effect in those studies including low-risk participants, the effect of including participants in whom no change in treatment would be expected.

Trends towards small reductions in cholesterol and blood pressure were also seen in studies in which risk information was only provided to patients. This highlights the central role patients have in making decisions about their treatment and the impact their risk perception and views about preventive medicine have on the outcomes: while some may be risk averse and start medication at low-risk levels, we know from existing literature that many patients are reluctant to start medication with 5% of people on the streets of London stating that they would not take a statin even if it gave them another 5 years of life,^47^ and people responding to a US-based internet survey prepared to pay an average of $1445 (£948; €1265) to avoid taking one pill a day for CVD prevention.^48^ The impact of provision of risk information is, therefore, complex and is likely to reflect a combination of factors from initial risk perception, peer comparison, beliefs about the disease, and to-date unmeasured effects such as medication adherence.

### Unanswered questions and future research

While this review shows that provision of risk information to patients and physicians improves accuracy of risk perception, increases prescribing and reduces levels of CVD risk factors and modelled CVD risk without causing changes in psychological well-being or anxiety, no studies included actual CVD events as an outcome, and so we are unable to comment on the effect of risk information on CVD morbidity or mortality. Additionally, while there is some suggestion that the effects may be greater in those at higher risk, with such a small number of studies that could simply reflect regression to the mean. There is, therefore, a need for further research with adequately powered trials with objective outcomes and longer follow-up to understand how best to communicate risk information to increase understanding, enhance shared decision-making and encourage behaviour change.

We are also unable to comment on the cost-effectiveness of provision of risk information and whether risk information delivered directly to patients is comparable to physician-led risk assessment. In the current age of increasing demand for healthcare and rising costs of treatment, ways of stratifying the population to enable delivery of interventions to those most likely to benefit are of increasing interest, but with such small changes observed in these studies, the question remains as to whether greater benefit could be derived from investment in population-wide prevention strategies^49^ rather than screening and individual risk assessment.
